# Cross-amplification and characterization of microsatellite loci for the Neotropical orchid genus *Epidendrum*

**DOI:** 10.1590/S1415-47572009005000037

**Published:** 2009-03-27

**Authors:** Fábio Pinheiro, Clarisse Palma-Silva, Fábio de Barros, Salvatore Cozzolino

**Affiliations:** 1 Departamento de Botânica, Instituto de Biociências, Universidade de São Paulo, São Paulo, SPBrazil; 2 Departamento de Botânica, Instituto de Biologia, Universidade Federal do Rio de Janeiro, Rio de Janeiro, RJBrazil; 3 Instituto de Botânica, Seção de Orquidário do Estado, São Paulo, SPBrazil; 4 Dipartimento di Biologia Strutturale e Funzionale, Complesso Universitario di Monte S. Ângelo, Università degli Studi di Napoli Federico II, NapoliItaly

**Keywords:** * Epidendrum*, Orchidaceae, short tandem repeat, cross-amplification

## Abstract

In this study we tested the cross-amplification of 33 microsatellite loci previously developed for two closely related Neotropical orchid genera (*Epidendrum* and *Laelia*). A set of ten loci were polymorphic across five examined species (20 individuals each) with 2 to 15 alleles per locus. The mean expected and observed heterozygosity (average across species) ranged from 0.34 to 0.82 and from 0.27 to 0.85, respectively. In addition we tested all loci in 35 species representative of the genus *Epidendrum*. Of these, 26 loci showed successful amplification. Cross-application of these loci represent a potential source of co-dominant markers for evolutionary, ecological and conservation studies in this important orchid genus.

*Epidendrum* L. is the largest of the orchid genera in the Neotropical region, with approximately 1500 species (Hágsater and Arenas, 2005). Species of this genus display extensive variation in morphological features and growth habits (epiphytic, lithophytic and terrestrial). Moreover, they offer an interesting opportunity for exploring the influence of human activities on natural environments, since they occur within several types of threatened vegetation (Amazon and Atlantic Rainforests, savannas, coastal sand dunes, ‘tepuis', and ‘páramos').

Microsatellite markers are the current choice for most studies on evolution, ecology and conservation, due to their high levels of polymorphism and high reproducibility. Studies increasingly aim at comparing genetic, demographic, behavioural, and breeding system parameters among related species. To address these questions, researchers require ‘universal' genetic markers that can easily be transferred between species (Barbara *et al.*, 2007). The capacity to transfer and apply the same set of microsatellite loci in different species can significantly facilitate studies among closely related and endemic taxa. This is important where resources for undertaking conservational genetic studies are limited, thus making it less cost-effective to develop specific microsatellite loci for many species of the same taxa.

Although, positive cross-amplification of some microsatellite loci have been previously reported for a few species of *Epidendrum* (Cortés-Palomec *et al.*, 2008; Pinheiro *et al.*, 2008a, 2008b), to date, there is no precise and standardized information about amplification-efficiency or polymorphism in these loci. The aim of this study is to report the potential of cross-species transferability of microsatellite markers across the genus *Epidendrum* in order to identify a set of polymorphic loci available for inquiries assessing the effect of landscape fragmentation on gene flow, species delimitation, origin and the maintenance of reproductive barriers among species of this genus.

Total genomic DNA was extracted from silica gel-exsiccated leaves according to the Pinheiro *et al.* (2008a) protocol. We tested three sets of microsatellite markers previously described for *Epidendrum fulgens* (nine loci - Pinheiro *et al.*, 2008a), *E. puniceoluteum* (ten loci - Pinheiro *et al.*, 2008b) and *Laelia speciosa* (14 loci - Cortés-Palomec *et al.*, 2008), the latter a closely related genus belonging to the subtribe Laeliinae, the same subtribe as that of *Epidendrum* (Hágsater and Arenas, 2005). Altogether, we tested 33 microsatellite loci (Table S4) for cross-amplification in 35 species belonging to different sections of genus *Epidendrum*.

For each microsatellite locus, the forward primers were synthesized with a 5'-M13 tail according to the Schuelke (2000) method, involving three primer polymerase chain reactions (PCRs), including a universal M13 primer labelled with a fluorescent dye, 6-FAM (Applied Biosystems). All PCR amplifications were performed in an Applied Biosystems 2700 thermocycler according to Pinheiro *et al.* (2008a, 2008b). The conditions were maintained constant for all loci so as to maximize standardization. Microsatellite alleles were resolved on a 3130 Genetic Analyzer (Applied Biosystems) and sized in accordance with LIZ (500) standard by using GENEMAPPER v. 3,7 software (Applied Biosystems).

We initially tested the potential of cross-amplification for all loci with one sample from each of the 35 *Epidendrum* species. Furthermore, we focused our effort on five of those species belonging to different phyletic sections of the genus *Epidendrum* (Hágsater and Arenas, 2005): *E. denticulatum, E. secundum, E. campestre, E. densiflorum* and *E. rigidum*. We sampled 20 individuals from each species of a single population (Table S1). GENEPOP software (Raymond and Rousset, 1995; web version 3.4) was used to calculate observed (*H*_O_) and expected (*H*_E_) heterozygosity, and to test for departure from Hardy - Weinberg equilibrium (HWE) as well as for linkage disequilibrium at each locus, by applying the Bonferroni correction to account for multiple comparisons.

Among the 33 loci tested, 26 showed positive amplification and PCR products with the expected allele sizes throughout most of the 35 species tested (Table S2). The percentage of cross-amplification was 78% on an average, thus higher than the mean value reported for monocot species (60% - Barbara *et al.*, 2007).

A total of ten polymorphic loci exhibited the features so desired for use as co-dominant molecular markers in the five examined species (Table 1), with the number of alleles per locus ranging from two to 15 (overall mean 5.6 alleles) Expected and observed heterozygosity ranged from 0.34 to 0.82 and 0.27 to 0.85, respectively (an average of 0.60 and 0.53, respectively) (Table 1). For each sampled population of the five species, we found sporadic cases of departure from HW equilibrium (p < 0.05): for loci EPP8 (in *E. denticulatum* and *E. rigidum*), EPP49 (in *E. densiflorum*), EPP56 (in *E. rigidum* and *E. secundum*), EPP86 (in *E. rigidum*), EFF45 (in *E. rigidum*), Lspe-1 (in *E. rigidum*) and Lspe-3 (in *E. rigidum*)*.* Interestingly, six out of ten loci in *E. rigidum* departed significantly from HW equilibrium due to heterozygotic deficiency. Such deviations could be caused by inbreeding and/or Wahlund effects arising from secondary population subdivision. Although null alleles cannot be ruled out, there was no evidence of scoring error due to ‘stuttering' or ‘large allele dropout', when using MICRO-CHECKER software (van Oosterhout *et al.*, 2004. Three loci in *E. campestre*, five loci in *E. denticulatum,* five loci in *E. rigidum* and seven in *E. secundum* exhibited linkage disequilibrium (p < 0.001)*.* Loci that were monomorphic or not amplified in most of the five *Epidendrum* species are listed in Table S3.

This study unveiled evidence that cross-transferability of developed microsatellite loci can increase the availability of markers to address both ecological and evolutionary questions in *Epidendrum*. The markers tested here showed to be of great potential for the use in comparing multiple co-occurring *Epidendrum* species in different ecological communities, thus contributing to knowledge on diversification processes and conservation among neotropical orchids.

## Figures and Tables

**Table 1:** Size range of the PCR products, number of observed alleles (A), expected and observed heterozygosity (*H*_*E*_/*H*_*O*_), and the significance of the test for departure from Hardy-Weinberg equilibrium, for the ten selected microsatellite loci (indicated by rows) in each of the five *Epidendrum* species. The size range of the original alleles described by the authors is indicated in parentheses below each locus.

Locus	Species	size range	A	*H*_*E*_/*H*_*O*_
EPP08	*E. campestre*	211-219	3	0.19/0.20
(219-223)	*E. densiflorum*	211-229	5	0.71/0.36
	*E. denticulatum*	211-213	2	0.51/1.00*
	*E. rigidum*	201-215	4	0.64/0.05*
	*E. secundum*	213-221	4	0.28/0.30
	Mean		3.6	0.47/0.38

EPP18	*E. campestre*	274-314	11	0.88/0.89
(288-324)	*E. densiflorum*	284-290	3	0.68/0.70
	*E. denticulatum*	288-328	15	0.92/0.84
	*E. rigidum*	284-312	4	0.64/0.55
	*E. secundum*	284	monomorphic	-
	Mean		8.3	0.62/0.60

EPP49	*E. campestre*	no amplification	-	-
(182-186)	*E. densiflorum*	162-187	9	0.79/0.32*
	*E. denticulatum*	176-190	6	0.78/0.70
	*E. rigidum*	170-186	3	0.46/0.10
	*E. secundum*	176-186	6	0.83/0.63
	Mean		6	0.71/0.44

EPP56	*E. campestre*	136-154	3	0.14/0.10
(136-144)	*E. densiflorum*	148-152	2	0.10/0.10
	*E. denticulatum*	132-166	10	0.87/0.85
	*E. rigidum*	152-156	2	0.51/0.00*
	*E. secundum*	122-162	9	0.78/0.28*
	Mean		5.2	0.48/0.27

EPP86	*E. campestre*	217-231	8	0.83/0.85
(215-239)	*E. densiflorum*	217-227	6	0.81/0.80
	*E. denticulatum*	217-223	4	0.71/0.60
	*E. rigidum*	217-235	7	0.83/1.00*
	*E. secundum*	215-229	8	0.85/1.00
	Mean		6.6	0.81/0.85

EFF26	*E. campestre*	190-204	8	0.76/0.84
(199-205)	*E. densiflorum*	196-202	4	0.66/0.95
	*E. denticulatum*	164-202	5	0.64/0.65
	*E. rigidum*	196-204	5	0.66/0.75
	*E. secundum*	192-204	6	0.77/0.88
	Mean		5.6	0.70/0.81

EFF45	*E. campestre*	280-284	3	0.50/0.35
(288-294)	*E. densiflorum*	288-294	3	0.49/0.53
	*E. denticulatum*	278-294	5	0.32/0.25
	*E. rigidum*	288-340	5	0.81/0.35*
	*E. secundum*	288-294	4	0.67/0.55
	Mean		4	0.56/0.41

EFF58	*E. campestre*	210-212	2	0.46/0.68
(210-212)	*E. densiflorum*	210-216	4	0.66/0.95
	*E. denticulatum*	212	monomorphic	-
	*E. rigidum*	210-212	2	0.51/1.00
	*E. secundum*	212	monomorphic	-
	Mean		2.7	0.34/0.54

Lspe-1	*E. campestre*	219-221	2	0.36/0.35
(350-390)	*E. densiflorum*	215-225	4	0.49/0.35
	*E. denticulatum*	471-493	3	0.34/0.28
	*E. rigidum*	225-233	2	0.49/0.00*
	*E. secundum*	462-488	9	0.85/0.85
	Mean		4	0.51/0.37

Lspe-3	*E. campestre*	250-266	8	0.83/0.79
(224-250)	*E. densiflorum*	250-288	12	0.88/0.40*
	*E. denticulatum*	262-304	15	0.93/1.00
	*E. rigidum*	268-286	5	0.78/0.25*
	*E. secundum*	244-260	6	0.69/0.90
	Mean		9.2	0.82/0.67

Significant departures from HWE: *p < 0.05.

## Supplementary Material

The following online material is available for this article:

Table S1 Geographical region, sample size and Biome of species sampled.

Table S2 Cross-species amplification of 26 loci tested for 30 additional *Epidendrum* species. Size range of the PCR products and unsuccessful amplifications are indicated (-).

Table S3 Size range of the PCR products, number of observed alleles (A), expected heterozygosity (He), observed heterozigosity (Ho), and the significance of the test for departure from Hardy - Weinberg equilibrium (HWE - Significant departures from HWE: p < 0,001), for the microsatellite loci (indicated by rows) that were not detected as polymorphic (monomorphic), or not amplified (na) in most of the five *Epidendrum* species. The size range of the original alleles described by the authors is indicated in parentheses on the bottom of each locus.

Table S4 Primer names, sequences and Genbank Accession numbers of 33 orchid species SSR loci.
