# Supplementary material for: Cross-amplification and characterization of microsatellite loci for the Neotropical orchid genus Epidendrum
Source: Genet Mol Biol. 2009 Mar 27;32(2):337–9. doi: 10.1590/S1415-47572009005000037 (PMC3036911; doi:10.1590/S1415-47572009005000037)
Supplement: Table S1 — Geographical region, sample size and Biome of species sampled. [file gmb-32-2-337-suppl1.pdf]

Table S1. Geographical region, sample size and Biome of species sampled.

**Species**	**N**	**Population**	**Biome**
E. denticulatum	20	Araruama, Rio de Janeiro, RJ	Atlantic Rainforest
E. densiflorum	20	Ilha do Cardoso, Cananéia, SP	Atlantic Rainforest
E. rigidum	20	Ilha do Cardoso, Cananéia, SP	Atlantic Rainforest
E. secundum	20	Serra do Cipó, Conceição do Mato Dentro, MG	Cerrado
E. campestre	20	Serra do Cipó, Conceição do Mato Dentro, MG	Cerrado
