# Supplementary material for: Cross-amplification and characterization of microsatellite loci for the Neotropical orchid genus Epidendrum
Source: Genet Mol Biol. 2009 Mar 27;32(2):337–9. doi: 10.1590/S1415-47572009005000037 (PMC3036911; doi:10.1590/S1415-47572009005000037)
Supplement: Table S2 — Cross-species amplification of 26 loci tested for 30 additional Epidendrum species. Size range of the PCR products and unsuccessful amplifications are indicated (-). [file gmb-32-2-337-suppl2.pdf]

Table S2. Cross-species amplification of 26 loci tested for 30 additional *Epidendrum* species. Size range of the PCR products and failed amplifications are indicated (-).

**Locus**	**epp_8§**	**epp_10§**	**epp_18§**	**epp_86§**	**epp_49§**	**epp_56§**	**epp_89§**	**epp_96§**	**eff_06Ψ**	**eff_26Ψ**	**eff_29Ψ**	**eff_43Ψ**	**eff_45Ψ**
species													
E. xanthinum	213	250-264	306-308	221-227	170-179	-	291	292-294	372	200-202	193	150	288
E. cattilus	214-216	306	280-290	219-241	156-165	119-146	-	-	-	200-202	198	150	286
E. calanthum	215	-	290-331	217-223	156-165	119	-	-	372	-	205	150	-
E. funkii	215-219	252-266	278	223-241	157-166	159	-	-	-	200-202	-	150	282-286
E. myrmecophorum	213-219	278	284-286	219-225	152-161	144-154	-	-	-	200-202	-	150	288
E. purpureum	213	268	284-300	241	-	141-153	289	300	370	198-	203	150	288
E. ibaguense	215	-	290-304	221-223	156-165	-	-	-	375	200-202	-	150	-
E. radicans	215	246	288-290	223-225	168-178	129-139	-	-	368	200-202	179-181	150	288
E. incisum	215	245	288-290	217-219	-	-	-	-	377	200-202	191	150	289
E. cinnabarinum	210	221	286	221-227	-	-	281-285	294-298	366-370	196-200	193-191	150	289
E. martianum	213-219	-	289-291	225	-	140-151	-	-	378	200-202	-	150	278
E. flexuosum	219	223	290	223	161-169	-	-	-	368	200-202	-	150	279
E. ramosum	213-219	250-254	290	219-223	152-160	140-151	-	-	-	200-202	-	150	288
E. saxatile	219	290	290	242	169-172	144-147	-	-	-	200-202	-	150	305-316
E. cristatum	215-219	236	284-286	219-225	-	133-144	-	-	372	200-202	-	150	289-291
E. purpurascens	219	254-261	304-306	225-227	169-172	-	-	-	-	200-202	-	150	-
E. ciliare	212-219	303,69	290-308	217-223	-	141-152	-	-	-	200-202	-	150	-
E. nocturnum	215-219	-	295-321	219-225	-	141-152	-	-	391	200-202	-	150	319-321
E. cooperianum	219	-	290	215-219	159-161	141-152	-	-	-	200-202	-	150	-
E. warasii	219	261	297	219-221	156-166	139-150	-	-	377	200-202	-	150	286
E. avicola	213-221	250-252	290-308	217-219	-	-	-	-	330	200-202	-	150	-
E. schlechterianum	212-219	290	290-308	219-227	167-169	241-246	-	-	-	200-202	-	150	-
E. coronatum	220-222	-	289-307	219	161-164	-	-	-	373	200	-	150	279
E. filicaule	219	-	290-306	225-229	139-149	-	-	-	377	200-202	-	150	-
E. chlorinum	219	-	290-306	217-219	-	-	259	-	-	200-202	-	150	321-323
E. tridactylum	212-219	-	290-306	-	188-198	-	-	-	-	200-202	-	150	-
E. vesicatum	215	-	290-308	227-229	166-168	-	-	-	-	200-202	-	150	295-301
E. latilabre	219-221	261	313	221-223	152-161	-	-	-	-	200-202	-	150	-
E. fulgens	210	265-275	286-308	219-243	152-160	-	284	-	366	199-203	201-211	150-152	291-295
E. puniceoluteum	211-219	271-273	290-310	221-227	160-184	136-144	288	294	370	197	219-225	150	289-291

§Markers isolated by Pinheiro et al (2008b); ΨMarkers isolated by Pinheiro et al (2008a); *Markers isolatated by Cortés-Palomec et al (2008).

Table S2. Continue

Locus	eff_58Ψ	eff_61Ψ	eff_70Ψ	eff_51Ψ	Lspe_1*	Lspe_3*	Lspe_4*	Lspe_6*	Lspe_8*	Lspe_9*	Lspe_10*	Lspe_11*	Lspe_14*
species													
E. xanthinum	212	265-271	329	335	218	249	214	172-175	221-247	219-229	-	205	254
E. cattilus	210	264	322-328	369-371	218-220	258-260	214	172-175	221	231-	-	205	254
E. calanthum	208	265	-	376-383	229	253	214	172-175	221	223-231	172-197	205	253
E. funkii	210	265	325-329	-	220	247	214	172-175	242	229-235	-	205	255
E. myrmecophorum	212-216	265	327-329	375-378	-	237-242	214	172-175	207-211	229-231	-	205	254
E. purpureum	212	265-270	328-341	375-377	215-218	233-237	214	172-175	207-211	232	172	205	254
E. ibaguense	208	265	300	-	218-220	269-271	214	172-175	208-210	231	172	205	254
E. radicans	210	265-272	308	374-376	221-227	258-260	214	172-175	243	231	-	205	252
E. incisum	210	266-275	329-331	376-382	242	241	214	172-175	221-247	229-233	-	205	254
E. cinnabarinum	210-212	266-270	325	376	-	-	214	172-175	244-246	232	-	205	252
E. martianum	210-215	262-265	320-325	-	218	260	214	172-175	226-230	229-231	197	205	258
E. flexuosum	208	265	336	-	225	250-252	214	172-175	-	231	275	205	253
E. ramosum	210-212	264-266	327-330	-	220	242	213-214	172-175	246	232	-	205	246
E. saxatile	210	266	339	-	226	234-236	214	172-175	203-205	229-231	196	205	209
E. cristatum	212	266	324-326	370	218-220	258	213	172-175	207	231	-	205	253
E. purpurascens	210	266	323-336	-	218	248-252	213-220	172-175	207-209	226-231	-	205	253
E. ciliare	222	264-266	325-329	339-340	218	259	214	172-175	214-218	227-232	219	205	259
E. nocturnum	211	264-266	329-336	372-380	217	239-242	214	172-175	220	227-233	-	205	253
E. cooperianum	216	-	323-326	373	219-222	261-263	213	172-175	237	231	-	205	253
E. warasii	216	264-266	318-330	372	218	264-272	214-218	172-175	219	231-237	196	205	246-253
E. avicola	210-213	264-266	323-325	272	-	253	213	172-175	220	232	-	205	254
E. schlechterianum	210	264-266	328	375	228-234	248	213-214	172-175	220	232	-	205	253
E. coronatum	210	262-266	318-323	373	226-228	242	213	172-175	238	231	152-172	205	249-256
E. filicaule	219	264-266	329-330	335	218	264-274	214	172-175	221	233-239	172	205	253
E. chlorinum	215	264-266	323-325	-	218	251	214	172-175	218-220	229-232	-	205	254
E. tridactylum	210-213	270-277	321-329	335	218-224	236	213	172-175	192	231-238	197-219	205	253
E. vesicatum	216	264-266	-	335	218-220	260-266	213	172-175	219-226	231	120-172	205	254
E. latilabre	210	264-266	328-338	374	216-226	268-270	213	172-175	247	231	-	205-209	253
E. fulgens	210-212	266	343-345	371-375	215	256-266	214	172-175	221-247	232	142	205	253
E. puniceoluteum	210-212	264-266	333-347	373-377	215-218	251-272	214	172-175	247	232	-	205	253

§Markers isolated by Pinheiro et al (2008b); ΨMarkers isolated by Pinheiro et al (2008a); *Markers isolatated by Cortés-Palomec et al (2008).

Su
