# Supplementary material for: Cross-amplification and characterization of microsatellite loci for the Neotropical orchid genus Epidendrum
Source: Genet Mol Biol. 2009 Mar 27;32(2):337–9. doi: 10.1590/S1415-47572009005000037 (PMC3036911; doi:10.1590/S1415-47572009005000037)
Supplement: Table S3 — Size range of the PCR products, number of observed alleles (A), expected heterozygosity (He), observed heterozigosity (Ho), and the significance of the test for departure from Hardy - Weinberg equilibrium (HWE - Significant departures from HWE: p < 0,001), for the microsatellite loci (indicated by rows) that were not detected as polymorphic (monomorphic), or not amplified (na) in most of the five Epidendrum species. The size range of the original alleles described by the authors is indicated in parentheses on the bottom of each locus. [file gmb-32-2-337-suppl3.pdf]

Table S3. Size range of the PCR products, number of observed alleles (A), expected heterozygosity (He), observed heterozigosity (Ho) and the significance of the test for departure from Hardy–Weinberg equilibrium (HWE - Significant departures from HWE: P< 0,001), for the microsatellite loci (indicated by rows) that were not successful polymorphic (monomorphic), or not amplified (na) in most of the five *Epidendrum* species. The size range of the original alleles described for the authors is indicated in parentheses on the bottom of each locus.

**Locus**	***Species***	**size range**	**A**	**He**	**Ho**	**HWE**
EPP10	*E. campestre*	na	-	-	-	-
(234–250)	*E. densiflorum*	na	-	-	-	-
	E. denticulatum	252-274	9	0.836	0.900	ns
	E. rigidum	na	-	-	-	-
	E. secundum	246-278	10	0.903	0.833	***
EPP89	*E. campestre*	na	-	-	-	-
(284-290)	*E. densiflorum*	na	-	-	-	-
	E. denticulatum	279-291	6	0.200	0.432	***
	E. rigidum	na	-	-	-	-
	E. secundum	na	-	-	-	-
EPP96	*E. campestre*	286-302	5	0.643	0.765	ns
(291-299)	*E. densiflorum*	na	-	-	-	-
	E. denticulatum	282-310	9	0.849	0.500	***
	E. rigidum	na	-	-	-	-
	E. secundum	286-308	8	0.795	0.450	***
EFF29	*E. campestre*	na	-	-	-	-
(185–229)	*E. densiflorum*	na	-	-	-	-
	E. denticulatum	193-225	13	0.920	0.850	***
	E. rigidum	na	-	-	-	-
	E. secundum	na	-	-	-	-
EFF43	*E. campestre*	150	monomorphic	-	-	-
(148–160)	*E. densiflorum*	150	monomorphic	-	-	-
	E. denticulatum	150-154	3	0.405	0.400	***
	E. rigidum	150	monomorphic	-	-	-
	E. secundum	150	monomorphic	-	-	-
EFF70	*E. campestre*	353-369	7	0.800	0.800	ns
(321–349)	*E. densiflorum*	na	-	-	-	
	E. denticulatum	na	-	-	-	
	E. rigidum	na	-	-	-	
	E. secundum	na	-	-	-	

Significant departures from HWE: ns – not significant; ***$P < 0.05$.

Table S3. Continued.

Locus	*Species*	size range	No. of alleles	He	Ho	HWE
Lspe-4	*E. campestre*	213	monomorphic	-	-	-
(176–189)	*E. densiflorum*	212	monomorphic	-	-	-
	E. denticulatum	213	monomorphic	-	-	-
	E. rigidum	na	-	-	-	-
	E. secundum	213	monomorphic	-	-	-
Lspe-6	*E. campestre*	na	-	-	-	-
(176–185)	*E. densiflorum*	172	monomorphic	-	-	-
	E. denticulatum	172	monomorphic	-	-	-
	E. rigidum	173-175	2	0.097	0.100	ns
	E. secundum	172	monomorphic	-	-	-
Lspe-8	*E. campestre*	na	-	-	-	-
(222–239)	*E. densiflorum*	na	-	-	-	-
	E. denticulatum	247-251	5	0.773	0.150	***
	E. rigidum	246	monomorphic	-	-	-
	E. secundum	na	-	-	-	-
Lspe-9	*E. campestre*	229	monomorphic	-	-	-
(190–206)	*E. densiflorum*	219-235	6	0.767	0.400	-
	E. denticulatum	na	-	-	-	-
	E. rigidum	na	-	-	-	-
	E. secundum	na	-	-	-	-
Lspe-11	*E. campestre*	204	monomorphic	-	-	-
(183–184)	*E. densiflorum*	204	monomorphic	-	-	-
	E. denticulatum	204	monomorphic	-	-	-
	E. rigidum	204	monomorphic	-	-	-
	E. secundum	204	monomorphic	-	-	-
Lspe-14	*E. campestre*	208	monomorphic	-	-	-
(221–233)	*E. densiflorum*	243-251	5	0.792	0.368	***
	E. denticulatum	252	monomorphic	-	-	-
	E. rigidum	259	monomorphic	-	-	-
	E. secundum	252	monomorphic	-	-	-

Significant departures from HWE: ns – not significant; ****P* < 0.05.
