# Supplementary material for: Cross-amplification and characterization of microsatellite loci for the Neotropical orchid genus Epidendrum
Source: Genet Mol Biol. 2009 Mar 27;32(2):337–9. doi: 10.1590/S1415-47572009005000037 (PMC3036911; doi:10.1590/S1415-47572009005000037)
Supplement: Table S4 — Primer names, sequences and Genbank Accession numbers of 33 orchid species SSR loci. [file gmb-32-2-337-suppl4.pdf]

Table S4: Primer names, sequences and Genbank Accession numbers of 33 orchid species SSR loci.

Name	Forward sequence	Reverse sequence	Genbank Accession
epp_8§	F: TGTTCAAGAACAACATCGGACT	R: TCTTGCTGGTTGGCATTATCT	EU326290
epp_10§	F: GGAGGCCAATGTGATGAAAC	R: TCGAATAAGCTCCTGCATCC	EU326291
epp_12§	F: GTCGGTGAGGGTCCAGAAA	R: CACCATCTTCTCTCCCCTGAG	EU326292
epp_17§	F: AGCACATCCGGGCCTAACTA	R: TGCCTGGCATCCATAATGAC	EU326293
epp_18§	F: TGCATACGTAACAACTGGAGGT	R: GGAAGGTCATTCTAACCAGGAA	EU326294
epp_49§	F: GCAAAGGGAGACGATTTGAG	R: AGCATTTTTCGCCCTTAACA	EU326295
epp_56§	F: ACGCTCTTTGGCTGGAACT	R: CTCACATGCCTTTAGCCTCAC	EU326296
epp_86§	F: CAGCCTTTAGGCATTCTTGG	R: GCTCATTGGCCTTAGTGACC	EU326297
epp_89§	F: TTCTTGTTGTCGCCTTCGAT	R: TCAGAGAGCTCGTCCGACA	EU326298
epp_96§	F: TCTAACATGCGAAGGCAAAA	R: TTTGGTTGTTAAGCCCCATT	EU326299
eff_06Ψ	F: TCAAGCCTATCATAAGTGCTCCA	R: CCTTGTTGCAACTGGGTGTT	EU363791
eff_26Ψ	F: TGTCCTAAGTCAAGTGGGGTTT	R: TCCGAGTCTGTCGGTCTTTT	EU363792
eff_29Ψ	F: TCCGCTGATTTGAGTTTGCT	R: CTGGTCCCGTAAGATCAATCAC	EU363793
eff_43Ψ	F: TGCCCCACAGACAATTAAGC	R: CCTCGATGGAACCCCATAAT	EU363794
eff_45Ψ	F: TTGGGTTTCGTCTCACATCA	R: CCCTCAGTATCCGCCACTT	EU363795
eff_51Ψ	F: CTTGTCTACGTGAGGGCACTG	R: TCAACAACGTGAAAAGCCATC	EU363796
eff_58Ψ	F: TGAATGCTTATACTCTCCCATCA	R: AAGTGGCAAAGCACCATGTA	EU363797
eff_61Ψ	F: TGTCCCCTATATTCTGATGGTG	R: AGGGTTTTAGGTCAAAGTGCTC	EU363798
eff_70Ψ	F: CGCGAGATTGTTCCAAACC	R: GCTCCACGCAAAACCTTTTTA	EU363799
Lspe_1*	F: AGAGAAAGCCCTTGTGTTGG	R: TCAGCTCTTCCGATTCTGGT	EF439820
Lspe_2*	F: GCAGATCCCACCATGAACTC	R: AATGTTGGAAATCGGTAGCA	EF439821
Lspe_3*	F: GCTTCAAGCAAGTGCAGAAA	R: AAGACAGGCCAACCAGAGAA	EF439822
Lspe_4*	F: GCATCGTTGAAGTTGCCAAT	R: TTTAGGGATCACCACCTTGG	EF439824
Lspe_5*	F: CCCACACAACCCCTGAACTA	R: ATGATTCGGGTTGACGAAAG	EF439825
Lspe_6*	F: GAAGCCCGTCGTCAGAAGTA	R: AAAAGAAGACCCCGAGCCTA	EF439826
Lspe_7*	F: CTTGAGGTGGGGAGTGATGT	R: GGCTTAGCTGTTTGGAATCG	EF439827
Lspe_8*	F: AAGCTCCTAGTGCCTGCTTG	R: CATGTGGCTCTGGATTGTTG	EF439828
Lspe_9*	F: GGGGAAGAAATGCAAACTAGC	R: CACAGGCATACGCACACAT	EF439829
Lspe_10*	F: TGGTTTGCTAAGTATTTCTTCAAGTT	R: TATGCAAAGCTTCCCCAAGT	EF439830
Lspe_11*	F: TGAATGCAAAATCCAATTGCT	R: GAAATATTCAAGCACGATGATCC	EF439831
Lspe_12*	F: GGGGAAACAGAAGAAGGAAGA	R: AGACCTGGAGCAACTTTCCA	EF439832
Lspe_13*	F: TCTCTAAATACCATAAGTGGAGTGAAA	R: TTCAACCGAGAGCCACCTAC	EF439835
Lspe_14*	F: TGAGACGAAAAACCCATTCTT	R: GGTTACCAGCCATTCCCTTT	EF439836

§Markers isolated by Pinheiro et al (2008b); ΨMarkers isolated by Pinheiro et al (2008a); *Markers isolatated by Cortés-Palomec et al (2008).
